# Substrate properties modulate cell membrane roughness by way of actin filaments

**DOI:** 10.1038/s41598-017-09618-y

**Published:** 2017-08-22

**Authors:** Chao-Hung Chang, Hsiao-Hui Lee, Chau-Hwang Lee

**Affiliations:** 1 Research Center for Applied Sciences, Academia Sinica, Taipei, 11529 Taiwan; 2 Department of Life Sciences & Institute of Genome Sciences, National Yang-Ming University, Taipei, 11221 Taiwan; 3 Institute of Biophotonics, National Yang-Ming University, Taipei, 11221 Taiwan; 4 Department of Physics, National Taiwan University, Taipei, 10617 Taiwan

## Abstract

Cell membrane roughness has been proposed as a sensitive feature to reflect cellular physiological conditions. In order to know whether membrane roughness is associated with the substrate properties, we employed the non-interferometric wide-field optical profilometry (NIWOP) technique to measure the membrane roughness of living mouse embryonic fibroblasts with different conditions of the culture substrate. By controlling the surface density of fibronectin (FN) coated on the substrate, we found that cells exhibited higher membrane roughness as the FN density increased in company with larger focal adhesion (FA) sizes. The examination of membrane roughness was also confirmed with atomic force microscopy. Using reagents altering actin or microtubule cytoskeletons, we provided evidence that the dynamics of actin filaments rather than that of microtubules plays a crucial role for the regulation of membrane roughness. By changing the substrate rigidity, we further demonstrated that the cells seeded on compliant gels exhibited significantly lower membrane roughness and smaller FAs than the cells on rigid substrate. Taken together, our data suggest that the magnitude of membrane roughness is modulated by way of actin dynamics in cells responding to substrate properties.

## Introduction

The plasma membrane roughness can be defined as the deviation of the actual membrane surface topography from an ideal atomically smooth surface. In recent years, cell membrane roughness has been proposed as a sensitive property to reflect various cellular physiological conditions. For example, Wang *et al*. proposed to use the membrane roughness as a visual diagnostic parameter to evaluate the efficacy of anti-cancer drug colchicine or cytarabine on various cancer cell lines^1^. Buys *et al*. found that the membrane roughness of red blood cells is decreased in diabetic patients, which could be related to the glycosylation or damage of the cytoskeleton proteins^2^. Pan *et al*. found that amyloid-beta 42 reduced the membrane roughness of neuroblastoma cells, and electric fields could remove this effect^3^. On the other hand, Yang *et al*. reported that N-methyl-D-aspartate treatment could increase the surface roughness and stiffness of neuroblastoma cells^4^. With various mechanisms, these previous studies showed that cell membrane roughness could be useful for judging the efficacy of specific stimulations presented in the microenvironment.

To date, atomic force microscopy (AFM) is the major tool to measure membrane roughness on living cells. However, in most cases the results obtained by AFM represent the mechanical properties of the membrane–cytoskeleton complex^5, 6^, even including the substrate properties^7^. Therefore, a comprehensive modelling is generally required to interpret the membrane roughness data obtained by AFM. In addition, because the image acquisition time of AFM is typically tens of minutes for a field of view containing a single cell, using AFM to accumulate statistically significant data could be very time-consuming. We proposed to use a wide-field optical technique named non-interferometric wide-field optical profilometry (NIWOP) to measure the membrane topography on living cells^8–11^. A NIWOP frame could be acquired within five seconds, and therefore NIWOP is also capable of conducting time-lapse studies on membrane dynamics. Because the NIWOP measurement is non-contact and label-free, it is useful for quantifying membrane roughness of living cells under various treatments^3, 12^.

For the membrane roughness measurements conducted on cells cultured on flat substrates, a fundamental question about the data interpretation is how the substrate properties influence the membrane roughness. It is well known that cells sense the substrate properties through the constituent proteins in focal adhesions (FAs)^13^. The substrate properties therefore modulate cytoskeleton structures through the cytoskeleton–FA linking components, such as talin or vinculin. We thus hypothesized that, when some mechanosensitive proteins in the FAs sense the differences in substrate properties and change the fine structures of cytoskeletons, the membrane roughness could reflect these responses.

In the present study, we measured the membrane roughness of mouse embryonic fibroblasts (MEFs) on various substrates using the NIWOP technique. We coated the substrate with different concentrations of fibronectin (FN) and correlated the sizes of FAs with the membrane roughness. We found that both the FA size and the membrane roughness increased with the FN concentration. We also used AFM to verify the dependence of membrane roughness on the FN concentration. When the polymerization of actin was inhibited, this correlation disappeared. In contrast, the structures of microtubules showed little relevance to the membrane roughness. Finally we used substrates of different rigidities to demonstrate the versatile sensitivities of membrane roughness to the culture environment.

## Results

### Membrane roughness and FA size on substrates with various concentrations of FN

**Figure 1:**
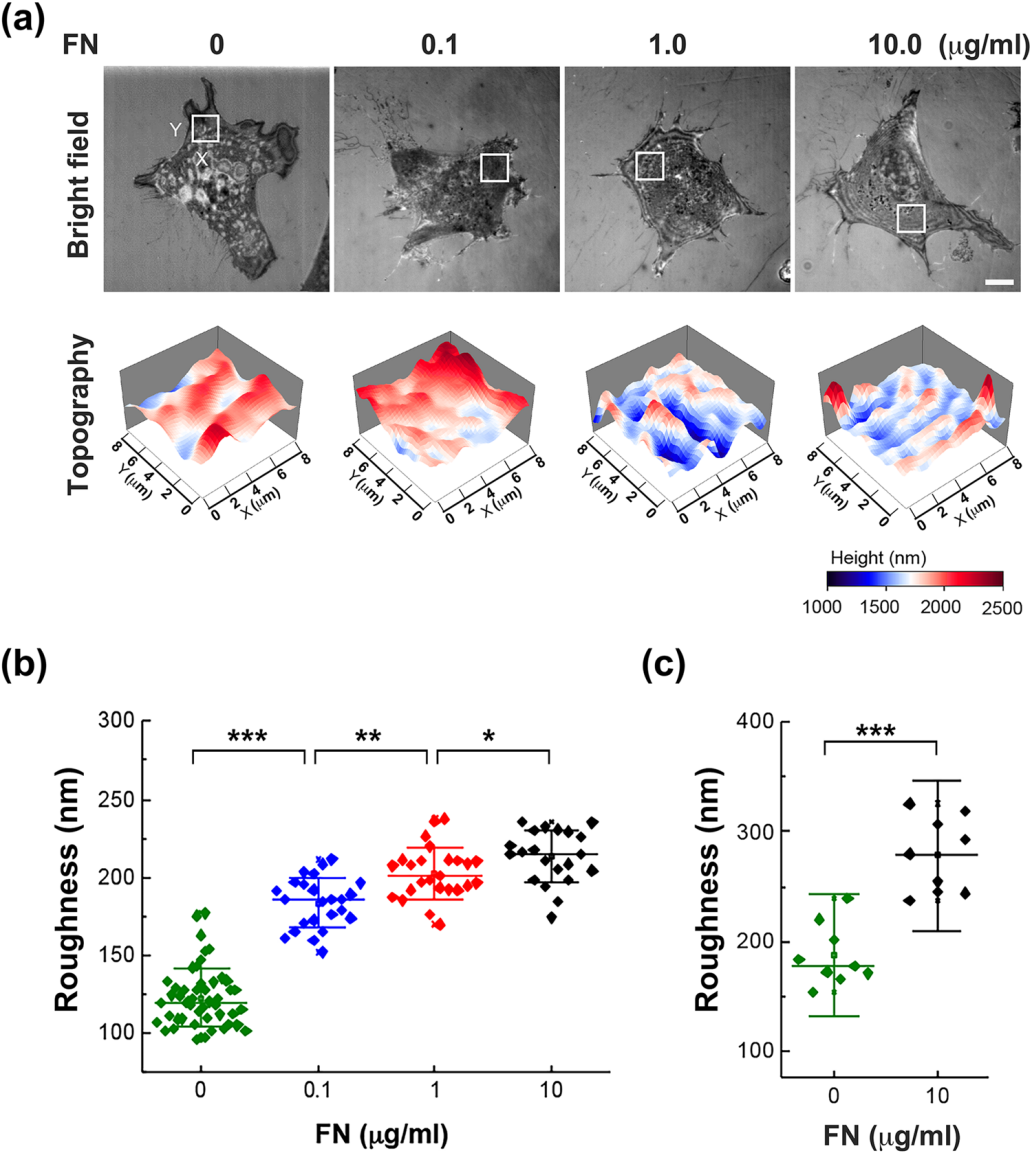
The membrane roughness is enhanced in cells responding to the increase of FN concentration on substrate surface. (**a**) Bright-field reflection image and the topography of MEFs determined by NIWOP. Cells were seeded on the polymer coverslip-bottom μ-dish coated with 0 to 10 μg/ml FN for 6 hours. In each condition, the region marked by the white square in the bright-field image is displayed in the membrane topography. Scale bar, 10 μm. (**b**) Membrane roughness of MEFs measured by NIWOP. (**c**) Membrane roughness of MEFs measured by AFM. Values represent mean ± standard deviation [*n* = 20–24 in panel (b); *n* = 9 in panel (c)]. *P < 0.05; **P < 0.01; ***P < 0.005 (Student’s t-test).

**Figure 2:**
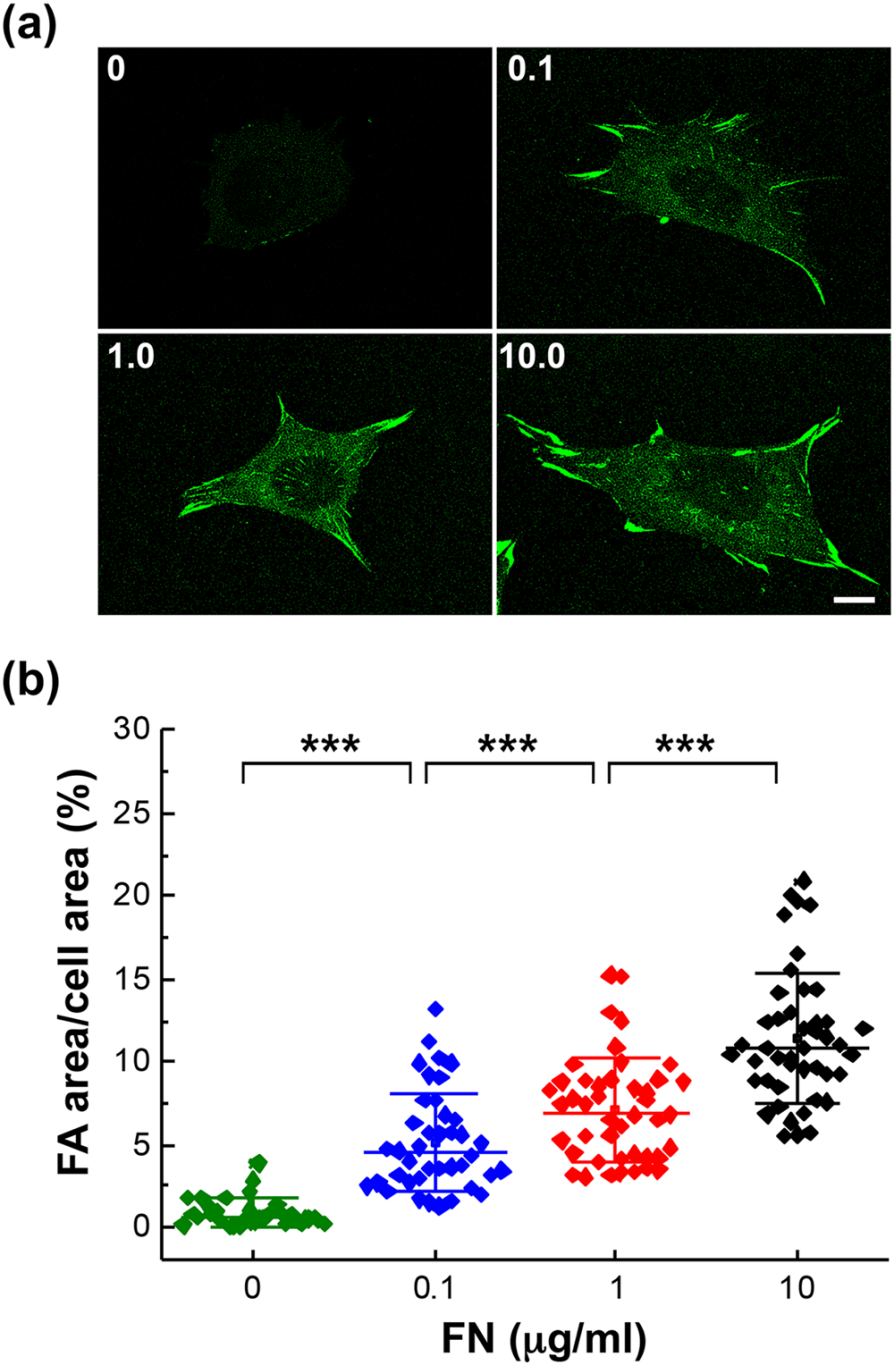
FA area is positively correlated with the FN concentration on substrate surface. (**a**) MEFs were seeded on the polymer coverslip-bottom μ-dishes coated with ploy-L-lysine followed by 0 to 10 μg/ml FN. After 6 hours, the cells were fixed and stained with anti-vinculin antibody for FA area determination. Scale bar, 10 μm. (**b**) The variation of FA areas in cells responding to various concentrations of FN. Data are expressed as the percentage of the total FA area of each cell relative to the cell area. Values represent mean ± standard deviation (*n* = 45 in each condition.). ***P < 0.005 (Student’s t-test).

It has been reported that the strength of cell–matrix adhesion could be enhanced by the increase of the concentration of extracellular proteins on substrate surface that affect the organization and activity of the actin cytoskeleton as well as FAs^14^. We first examined the membrane roughness of MEFs seeded on dishes coated with different concentrations of FN from 0 to 10 μg/ml. In order to reduce the possibility of non-specific binding on the commercial culture dish, all the dishes were coated with poly-L-lysine (PL) before the FN coating. Using NIWOP, we found that the membrane roughness was enhanced with the increase of the FN concentration (Fig. 1a and b). The results of the NIWOP measurement agreed with those obtained by AFM, verifying the reliability of our optical measurement (Figs. 1c and S1). We also determined the total FA area in each cell that can reflect the cell–matrix adhesion strength using immunostaining with anti-vinculin antibody. As expected, the FA area was increased with the FN concentration (Fig. 2). The correlation between the membrane roughness and the FA area implies that membrane roughness might be influenced by substrate properties through some signal transduction mechanisms related to the FAs.

### Membrane roughness and FA size under the alterations of actin filaments and microtubules

FAs provide the linkages to extracellular matrix (ECM) at the integrin binding sites and transmit mechanical forces between the ECM and the actin cytoskeleton^13, 15^. It is well known that the formation of FAs is usually coupled with the polymerization of actin filaments, and the contractility generated by actomyosin can regulate FA dynamics^16^. An enhancement in contractility promotes FA stabilization and maturation, while a reduction leads to FA disassembly^13^. The maturation of FAs also modulates actin assembly reciprocally^15, 17^. In addition to F-actin, microtubules have been observed to target peripheral FAs and to promote FA turnover^18, 19^. We thus postulated that the membrane roughness might be influenced by the status of cell cytoskeletons.

**Figure 3:**
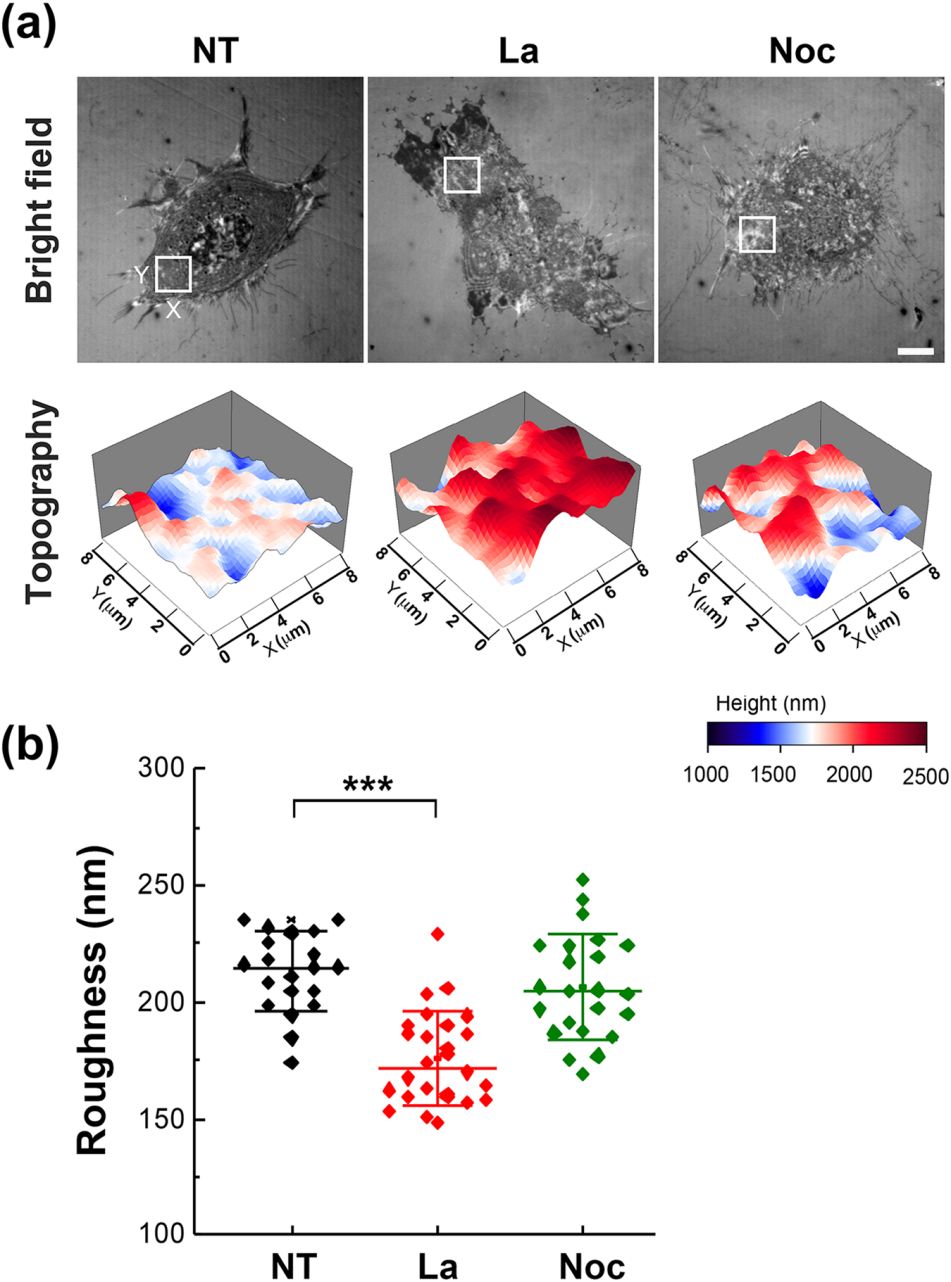
Membrane roughness under the alterations of cytoskeletons. (**a**) Bright-field reflection image and the topography of MEFs measured by NIWOP. Cells were seeded on the 10 μg/ml FN-coated polymer coverslip-bottom μ-dishes and treated with latrunculin (La; 1.0 μM) or nocodazole (Noc; 10 μM) for 30 min followed by the NIWOP measurement. NT, no treatment. In each condition, the region marked by the white square in the bright-field image is displayed in the membrane topography. Scale bar, 10 μm. (**b**) Membrane roughness of MEFs measured by NIWOP. Values represent mean ± standard deviation (*n* = 20–24). ***P < 0.005 (Student’s t-test).

In order to know the correlation between membrane roughness and actin filaments, cells seeded on 10 μg/ml FN-coated dishes were cultured with or without 1 μM of latrunculin to inhibit actin polymerization for 30 minutes, and then the membrane roughness was assessed by NIWOP. We found that the inhibition of actin polymerization significantly decreased the membrane roughness (Fig. 3). The reduction of actin filaments after the latrunculin treatment was confirmed by fluorescence staining with phalloidin (see Supplementary Fig. S2). The data in Fig. 3 suggest that actin remodeling should be required for the increase and maintenance of membrane roughness. On the other hand, microtubules targeting the matrix-contact sites have been known to mediate the disassembly of FAs; and the disruption of microtubules leads to the stabilization of FAs^17, 20^. To know whether the stability of microtubules is involved in the regulation of membrane roughness, cells seeded on FN-coated dishes were treated with 10 μM of nocodazole to disrupt microtubules (see Supplementary Fig. S2). We found that the disruption of microtubules had almost no effect on membrane roughness (Fig. 3). It seems that the maintenance of membrane roughness requires the polymerization or integrity of F-actin, but is independent of stabilized microtubules.

**Figure 4:**
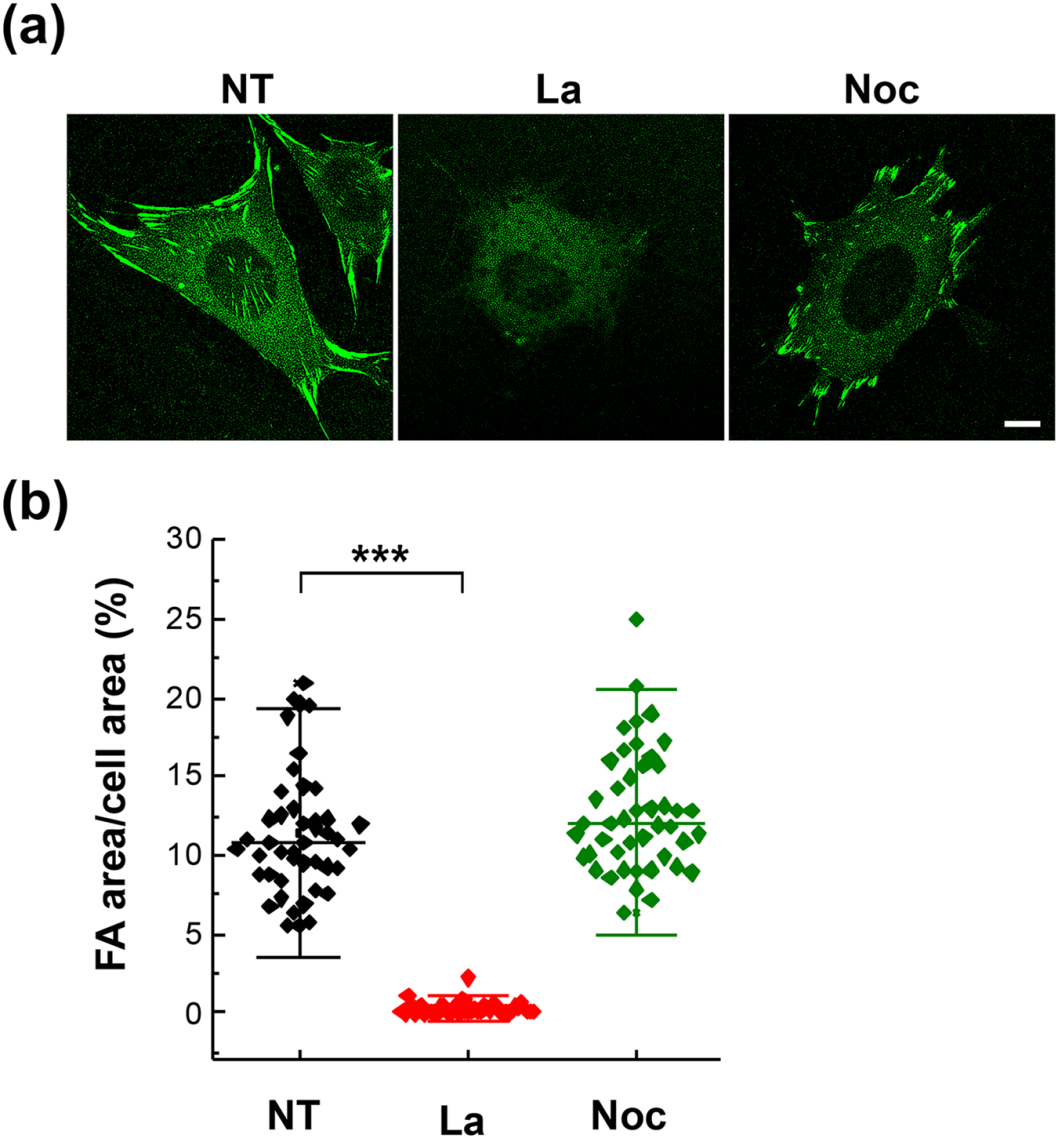
Actin polymerization is required for FA maturation. (**a**) MEFs were seeded on the 10 μg/ml FN-coated polymer coverslip-bottom μ-dishes and treated with latrunculin (La; 1.0 μM) or nocodazole (Noc; 10 μM) for 30 min followed by immunostaining with anti-vinculin antibody for FA area determination. NT, no treatment. Scale bar, 10 μm. (**b**) The variation of FA areas under various treatments. Data are expressed as the percentage of the total FA area of each cell relative to the cell area. Values represent mean ± standard deviation (*n* = 45). ***P < 0.005 (Student’s t-test).

We determined the FA areas of cells treated with latrunculin and nocodazole by immunostaining with anti-vinculin antibody. The treatment with latrunculin abrogated the formation of FAs, while the cells treated with nocodazole exhibited obvious FAs (Fig. 4). It has been shown that the FA serves as a signal center to sense and respond to the increase of ligand concentration, leading eventually to the strengthening of FAs and stress fibers^13, 15^. Hence we speculated that the disruption of FAs could be one of the reasons for the reduction of membrane roughness in the latrunculin-treated MEFs.

**Figure 5:**
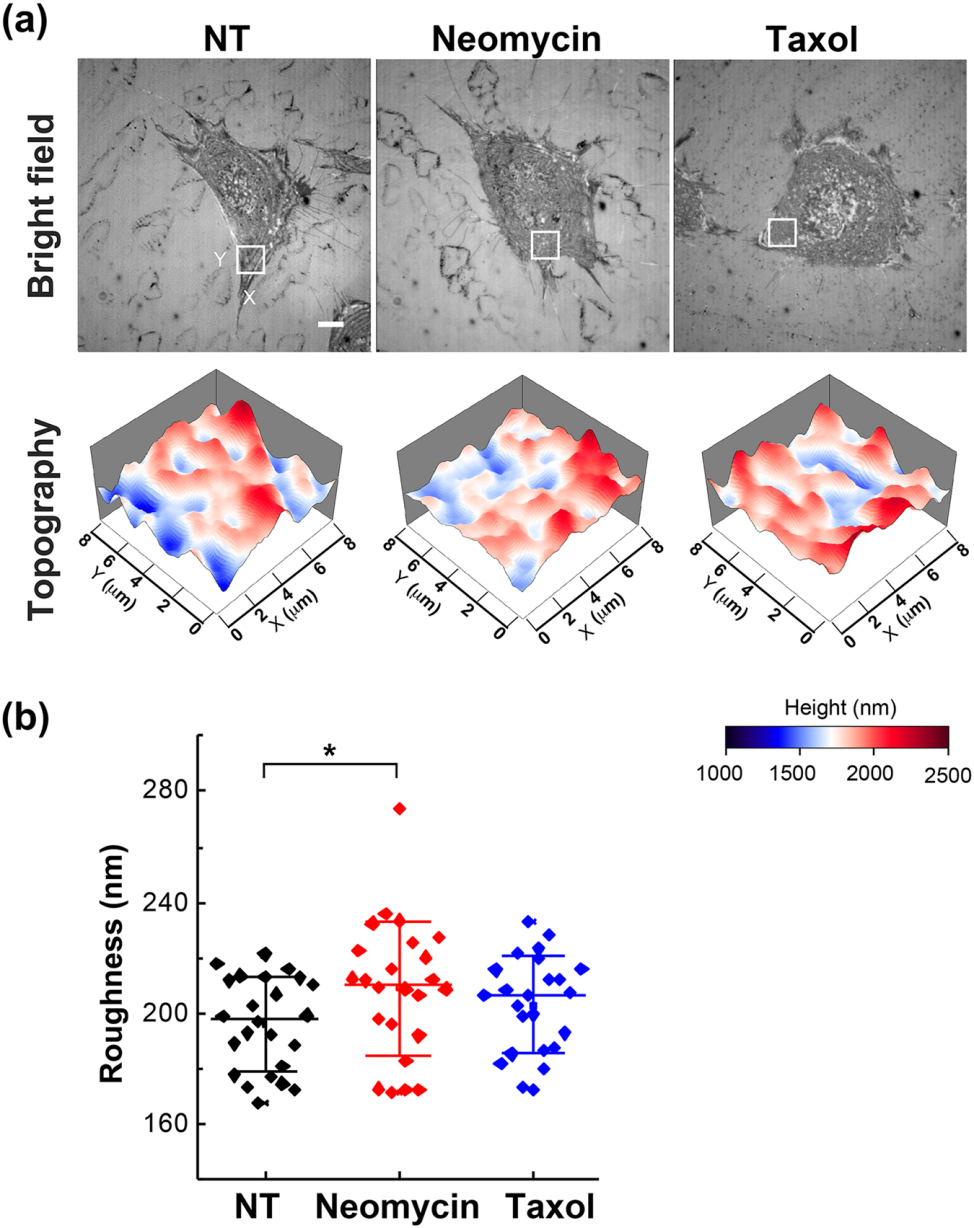
Membrane roughness of cells in response to cytoskeletal reagents. (**a**) Bright-field reflection image and the topography of MEFs measured by NIWOP. Cells were seeded on the 10 μg/ml FN-coated polymer coverslip-bottom μ-dishes and treated with neomycin (3 mM) for 4 hours or Taxol (10 μM) for 30 min followed by the NIWOP measurement. NT, no treatment. In each condition, the region marked by the white square in the bright-field image is displayed in the membrane topography. Scale bar, 10 μm. (**b**) Membrane roughness of MEFs measured by NIWOP. Values represent mean ± standard deviation. *P < 0.05 (Student’s t-test).

The formation of FAs is usually coupled with the actin polymerization, and the treatment of cells with latrunculin results in the disruption of both processes. To further examine whether the membrane roughness is modulated by way of actin filaments, we utilized neomycin, a reported inhibitor of talin-dependent integrin clustering that functions by masking membrane phosphatidylinositol 4,5-bisphosphate (PIP_2_)^21, 22^. The treatment of cells with neomycin results in the increased turnover of integrin as well as the potentiation of retrograde actin flows and the accumulation of peripheral actin-based structures^22–24^. As shown in Fig. 5, the neomycin treatment slightly increased the membrane roughness. By fluorescence staining with phalloidin, we detected intact F-actin in the neomycin-treated MEFs (see Supplementary Fig. S3). These data indicated that the actin cytoskeleton, in particular the peripheral actin filaments, played a major role in the regulation of membrane roughness. In other words, membrane roughness is modulated by way of actin filaments in cells responding to substrate properties. In our previous work, we showed that the membrane roughness of neuronal N2a cells was sensitive to the treatment of a microtubule-stabilization drug paclitaxel (Taxol)^12^. Here we also tested the effect of Taxol on the membrane roughness in MEFs and found that the Taxol treatment for 4 hours had no significant effect on the membrane roughness (Fig. 5). This is a reverse result to that obtained with N2a cells. Nonetheless, this result agreed with the finding that the microtubule disruption by nocodazole did not change the membrane roughness in MEFs (Fig. 3). Taken together, our data suggest that the increase of membrane roughness might be a representation of cell adhesion strength, which is regulated by the actin cytoskeleton.

### Membrane roughness is sensitive to the substrate rigidity

**Figure 6:**
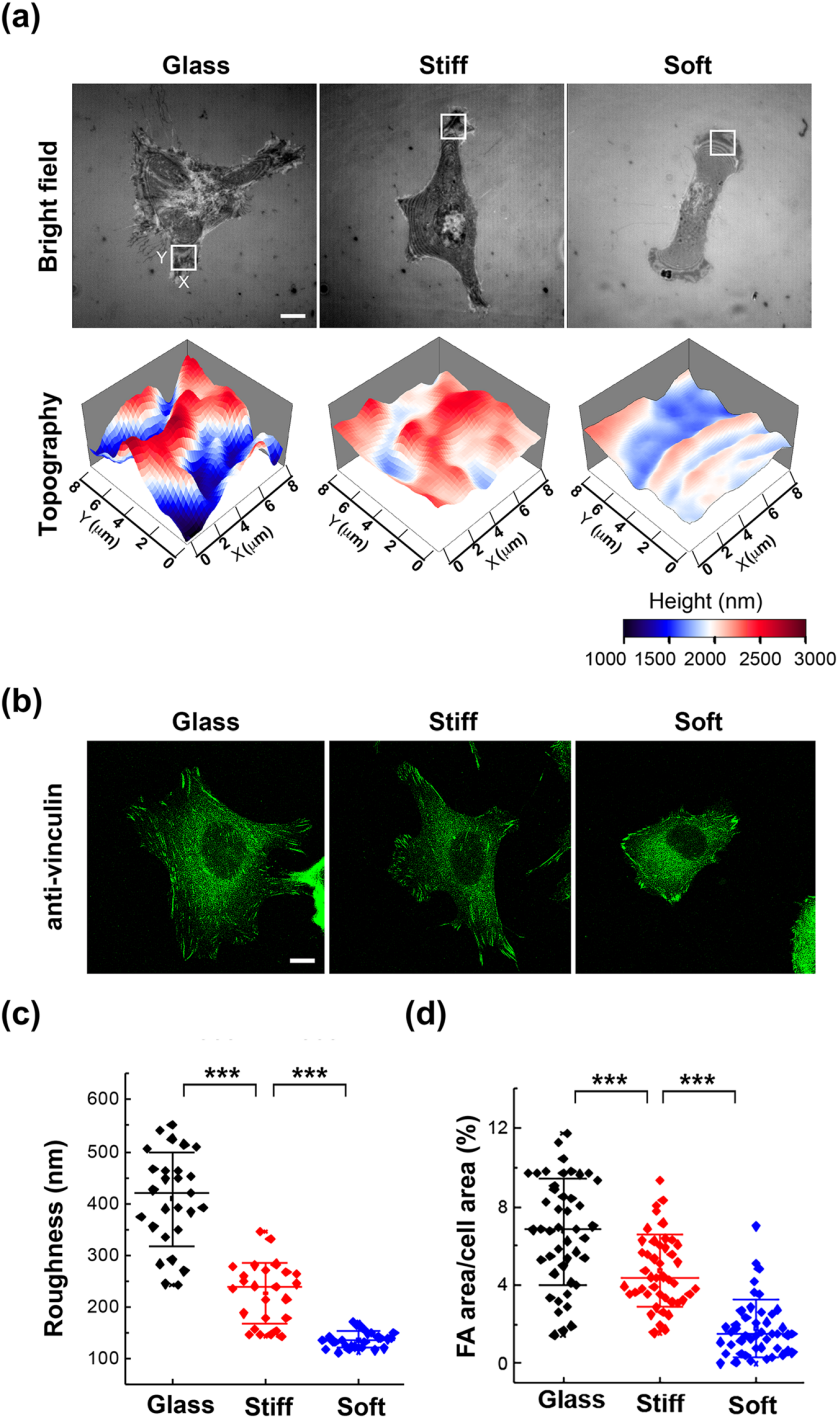
Membrane roughness is sensitive to the matrix rigidity. Cells were plated on the 10 μg/ml FN-coated glass coverslips or PAA gels with Young’s modulus of 24.2 kPa (stiff) and 3.7 kPa (soft) for 6 hours. (**a**) Bright-field reflection image and the topography of MEFs determined by NIWOP. In each condition, the region marked by the white square in the bright-field image is displayed in the membrane topography. Scale bar, 10 μm. (**b**) Cells were fixed and stained with anti-vinculin antibody for FA area determination. (**c**) Membrane roughness of MEFs measured by NIWOP. (**d**) The variation of FA areas on substrates of different magnitudes of rigidity. Data are expressed as the percentage of the total FA area of each cell relative to the cell area. Scale bars, 10 μm. Values represent mean ± standard deviation [*n* = 20–24 in panel (c); *n* = 45 in panel (d)]. ***P < 0.005 (Student’s t-test).

The ECM rigidity can be sensed by the force-sensing molecules in FAs that convert the physical signals into intracellular biochemical signals, which results in cell deformation by changing intracellular contractility through cytoskeletons^25, 26^. The rigidity of ECM plays pivotal roles in many cellular behaviors, such as durotaxis and cell differentiation^27, 28^. To know whether the membrane roughness is a type of rigidity response, we made two elastomeric polyacrylamide (PAA) gels and measured their magnitudes of rigidity by AFM, which were 3.7 kPa corresponding to the soft gel and 24.2  kPa for the stiff gel (see Supplementary Fig. S4). The gels were conjugated with 10 μg/ml of FN for cell adhesion. We found that cells seeded on the soft gel exhibited much lower membrane roughness than those on the stiff gel, and the cells on glass substrate had the highest membrane roughness (Fig. 6a and c). The fluorescence microscopy images also showed that the FA areas were positively dependent on the substrate rigidity (Fig. 6b and d). Therefore we ascertained that cell membrane roughness was sensitive to the substrate rigidity, partly by way of the FA sensing mechanisms.

## Discussion

The integrin-mediated adhesion signals influence many cellular processes, including cell growth, differentiation, migration, and gene expressions^28–30^. In the present study, we used the NIWOP measurement to address whether adhesion signals regulate the plasma membrane roughness. The results for cells seeded on dishes coated with different concentrations of FN as well as cells on PAA gels with different rigidities showed that the plasma membrane roughness was sensitive to substrate properties both chemically (the FN concentration) and physically (the substrate rigidity). The membrane roughness was enhanced when the ECM protein density or the substrate rigidity increased. This finding implies that membrane roughness is regulated by mechanosensitive events and might be involved in the relevant cellular processes, such as cell migration.

For cells well attaching on the substrate (e.g., the cells on substrate coated with 1 or 10 μg/ml FN), we could clearly observe periodical profiles, or waves, on the membranes. These membrane waves have been well described in the literatures and are believed to be closely related to cell motility^31–34^. In one of our previous publications^10^, we have measured the membrane waves on fibroblasts by using NIWOP, and fitted the amplitudes, wavelengths, and speeds to the active membrane wave model proposed by Shlomovitz and Gov^35^. However, in the active membrane wave model the involvement of FAs was not included. From the results in the present study, we confirmed that the properties of FAs could also be relevant to the periodical membrane profiles through the actin cytoskeleton. In other words, to understand the kinetics of membrane topography, we must also consider the properties of FAs and the adhesion signalling. But the detailed mechanosensing processes need to be investigated with more experiments.

The intrinsic deformability of the plasma membrane influences many cell processes, such as vesicle trafficking and cell motility^36–38^. The magnitude of membrane roughness probably depends on the membrane tension as well as the adjacent actin cytoskeletons and their connections to the membrans^31, 35^. Cell cortex is an actin-rich network consisting of F-actin, myosin motors, and actin-binding proteins on the inner face of the plasma membrane at cell periphery^39^. Cell cortex attaches to the plasma membrane via the ERM protein family (ezrin, radixin, moesin, plus merlin), which not only interacts with transmembrane proteins and cytoskeletons but also plays a crucial role in organizing membrane domains^40^. ERM proteins associate with integral protein of the plasma membrane via their conserved N-terminal FERM domain and with F-actin via the charged C-terminal domain^41, 42^. It has been reported that moesin also binds to and stabilizes microtubules via its FERM domain at the cell cortex^43^. In the present study, we found that membrane roughness was significantly reduced by the treatment with latrunculin but not nocodazole. This result suggests that the variation of membrane roughness in response to the substrate properties is mainly due to the contributions from actin dynamics.

In addition to the actin/membrane linker proteins, the levels of membrane PIP_2_ have been shown to control the adhesion of the membrane bilayer to the underlying cytoskeletons through the influence on integrin clustering^44, 45^. The treatment of cells with neomycin, an aminoglycoside antibiotic, inhibits the phosphatidylinositol cycle by selectively binding with PIP_2_ and leads to the reduction of integrin clustering as well as the potentiation of lamellipodium formation and ruffling activity^22, 46, 47^. Schulte *et al*. had showed that neomycin did not disturb the general cellular competence for lamellipodial actin polymerization, but accelerated the retrograde actin flow rates in HEK293 cells^23^. In the present study, the neomycin treatment also slightly enhanced the membrane roughness. This result indicated that the membrane/integrin/F-actin linkage was involved in the substrate dependency of membrane roughness. Additional experimental approaches such as knocking down the ERM proteins or overexpressing a talin variant that lacks the actin binding site to disrupt the membrane/integrin/F-actin linkage may provide interesting and clear information to better understand the mechanisms connecting substrate properties to membrane roughness.

In our previous works^3, 12^, we demonstrated that the membrane roughness of neuroblastoma cells is sensitive to various external stimulations, including amyloid peptides of different conformations, electric fields, an anti-cancer drug Taxol, gold nanoparticles, and a hypertonic solution. Nonetheless, the treatments used in those previous works were all solution-based. We did not consider the stimulations from the substrate. The hypothesis of the present work is that, the force and other adhesion signals from FAs could propagate through the cytoskeletal structure and affect membrane topography. Therefore the membrane roughness could be sensitive to the properties of the substrate. We also chose another cell type, fibroblasts, for the present study because the FA sizes of fibroblasts are much larger than those of neuronal cells.

Fluorescent protein-tagged paxillin and utrophin are widely used as molecular probes for imaging FAs and actin filaments, respectively^48, 49^. Here we provide evidence showing that the variations of plasma membrane roughness are correlated with FA areas and dependent on F-actin structures. The label-free advantage of NIWOP measurements can avoid artificial results caused by exogenous protein expressions in comparison with fluorescence imaging.

About the spatial dimension, researchers generally consider that the nanometer scale is important for elucidating the ligand–receptor interactions on cell membranes^50^. However, seeing membrane topography in various scales is useful for understanding various types of cellular mechanosignaling processes. The 100-nm to micrometer scale is suitable for studying cellular responses relevant to the configuration changes of cytoskeletons. In fact, a number of AFM studies on cell membrane roughness were focused on the 100-nm to micrometer scale^4, 51–53^. The depth sensitivity of NIWOP is particularly suitable for the measurement in this scale.

FAs link a cell to ECM at integrin binding sites and play dual roles in force transmission and signal transduction^13^. It seems generally accepted that cell adhesion causes extensive changes of the local compositions of plasma membranes, as a consequence of lateral movements of surface and cytoskeletal molecules^54^. Recently, integrins have been confirmed to mediate the rigidity sensing function of adherent cells^55^. Because of the connection between integrins and actin filaments, our findings in the current work suggest that membrane roughness should also be considered as a parameter correlated with the FA sensing functions. It was reported that, on the PAA gels of low stiffness (0.5 kPa), stem cell differentiation was affected owing to the porous substrate surface^56^. The mechanosensing mechanisms on nanotopography could be transduced by the integrin-activated focal adhesion kinase^57^. Recently, Schulte *et al*. used nanostructured substrate surface to demonstrate that the changes in FA areas affected cytoskeletal configurations and dynamics, which modulated the differentiation of PC12 cells^58^. We thus suspected that the differences in membrane roughness and FA areas on substrates of different magnitudes of rigidity might also be related to the surface nanotopography. In the present work, we checked the surface topography of the 3.7 kPa and 24.2 kPa PAA gels by SEM imaging (see Supplementary Fig. S5). Although the pore sizes on the surfaces of these two gels showed large variations, on the stiff gel we did observe more small pores (with diameters smaller than 1.0 μm) than on the soft gel. The small pores might be caused by more crosslinking of the PAA polymers or the collapse of structure during the substrate preparation for electron microscopy. Therefore we could not entirely exclude the influence on the membrane roughness and FA areas by the surface topography. More experiments with well-defined surface nanofeatures are required to deal with this consideration.

## Conclusion

In the present work, we demonstrated that cell membrane roughness was sensitive to the substrate properties and related to the status of FAs. On substrates coated with different concentrations of fibronectin and those with different magnitudes of rigidity, the areas of FAs showed positive correlations with the membrane roughness. Actin filaments connected FAs with membrane roughness. In contrast, disruption or stabilization of microtubules did not change the membrane roughness. Since the FA is the essential element for cells to sense substrate properties, we believe that the non-contact and label-free quantification of cell membrane roughness by NIWOP would be useful for fast diagnostics of cellular responses caused by chemical and physical stimulations that alter the conditions of FAs. In addition, because the NIWOP is an all-optical wide-field measurement technique, to acquire the membrane roughness data of hundreds of cells in a single experiment is feasible.

## Methods

### Cell culture

The wild type mouse embryonic fibroblasts (MEFs) were kindly provided by Gen-Sheng Feng, (Molecular Pathology Graduate Program, University of California, San Diego, USA) and cultured in Dulbecco’s Modified Eagle Medium (DMEM) containing 10% fetal bovine serum (FBS) and 1% antibiotic pen-strep-ampho. Before the NIWOP observation, cells were plated on polymer coverslip-bottom μ-dishes (ibidi GmbH, Martinsried, Germany) or glass coverslips coated with 10 μg/ml of poly-L-lysine and different concentrations of fibronectin (0–10 μg/ml) for 6 hours.

### Setup and operation of the NIWOP system

The NIWOP technique is based on the structured-illumination wide-field optical sectioning microscopy^59^ and differential confocal microscopy^60^. The apical surface of the cell to be observed was placed into the linear region of the axial response curve of the sectioning microscopy. The optical intensity measured in the linear region is proportional to the height of the sample. We used a calibration procedure to eliminate the reflection signal from the bottom surface of the cell^8^. In this way, NIWOP measures the membrane topography in the optical far field with nanometer height sensitivity. With a 40×, 0.80 numerical aperture objective lens as the probe, the NIWOP system provided a depth sensitivity about 52 nm, and a dynamic range about 3000 nm. The whole NIWOP system was placed in a temperature-controlled microscope cage that provided a constant-temperature environment (37 ± 0.5 °C) for the live-cell experiments. More details about the operation of the NIWOP system can be found in our previous publications^3, 9–12^.

We defined the membrane roughness as the standard deviation of membrane heights in an 8 × 8 μm^2^ region arbitrarily selected on the apical surface of a cell. We avoided the cell nucleus in the measurement because of its higher rigidity than that of the cytoplasm. Because the typical periods of the membrane ripples are around 1–4 μm (see the membrane topography in Figs 1, 3 and 5), we did not consider using an area smaller than 4 μm. For the simplicity of the data processing program, we used square areas. Experimentally we found that an 8 × 8 μm^2^ area was the most suitable size for the roughness quantification. In order to obtain general results, in one cell we selected 3–5 regions to collect the membrane profiles. For the data in one 8 × 8 μm^2^ region we calculated the standard deviation of all height values at every pixel, and then used the average of the standard deviations of the 3–5 regions as one data point of the cell. In this way we believed that the variations caused by sampling locations on the cell membrane should be averaged out.

### Immunofluorescence staining of the FA

For the measurement of FA areas, we fixed the cells by using 3.7% paraformaldehyde in phosphate buffered saline (PBS) for 30 minutes and then washed the cells three times with PBS. Cells were permeated with the permeation solution that contained 0.3% Triton X-100, 50 mM Tris-HCl pH 7.4, and 150 mM NaCl. Then we washed the cells with the TBST (Tris-buffered saline with Triton X-100) that contained 0.1% Triton X-100, 50 mM Tris-HCl pH 7.4, and 150 mM NaCl. The cells were blocked with 5.5% FBS in PBS for one hour at room temperature. We labeled the FAs by using the anti-vinculin antibody conjugate with Alexa Fluor^®^ 488 (53–9777, eBioscience, UK). The fluorescence images of FAs were acquired by using a confocal microscope (TCS-SP5, Leica Microsystems, Germany) with a 63×, 1.4 numerical aperture oil-immersion objective.

For the quantification of FA areas, we set a fixed threshold for all the experiments to identify the FAs from the background of the fluorescence images. Then we used the built-in function of ImageJ (http://rsb.info.nih.gov/ij/) to calculate the areas of the FAs automatically. The total FA area was then normalized to the individual cell area for comparison.

### Atomic force microscope measurement

We employed an atomic force microscope (NanoWizard^®^ 3 BioScience AFM, JPK Instruments AG, Germany) to observe the membrane topography on living MEFs. We used an AFM probe with a 125-μm long cantilever made of a quartz-like material (qp-SCONT, NANOSENSORS, NanoWorld AG, Switzerland). The spring constant of the cantilever was 0.01 N/m.

### Preparation of polyacrylamide gels

Elastic polyacrylamide (PAA) gels with different Young’s elastic moduli were prepared based on the method developed by Wang and colleagues^61^. The final concentrations of acrylamide/Bis-acrylamide were 5%/0.1% (vol/vol) for the soft gel and 10%/0.2% for the stiff gel. The gels coated on the glass coverslips were typically 30–40 μm thick. For cell adhesion, the bovine fibronectin (10 μg/mL) was covalently cross-linked with PAA surface by the bifunctional cross-linker N-sulfosuccinimidyl-6-[42-azido-22-ni- trophenylamino] hexanoate (sulfo-SANPAH, ProteoChem). The mechanical properties of the PAA gels were measured by AFM.

### Data analysis

In the present work, the experiment of each condition was repeated three times, and we show the data from all cells in the three repeats together. One dot in the data figures represents the average of roughness measured in 3–5 8 × 8 μm^2^ regions in one cell. Statistical significance was determined by using unpaired two-tailed Student’s t-test.

## Electronic supplementary material

Supplementary data
