# Supplementary material for: Substrate properties modulate cell membrane roughness by way of actin filaments
Source: Sci Rep. 2017 Aug 22;7:9068. doi: 10.1038/s41598-017-09618-y (PMC5567215; doi:10.1038/s41598-017-09618-y)
Supplement: Supplementary file 1 — Supplementary data [file 41598_2017_9618_MOESM1_ESM.pdf]

Supplementary Information

Substrate properties modulate cell membrane roughness by way of actin filaments

Chao-Hung Chang[1], Hsiao-Hui Lee[2*] and Chau-Hwang Lee[1,3,4*]

[1]Research Center for Applied Sciences, Academia Sinica, Taipei 11529, Taiwan.

[2]Department of Life Sciences & Institute of Genome Sciences, National Yang-Ming University, Taipei 11221, Taiwan.

[3]Institute of Biophotonics, National Yang-Ming University, Taipei 11221, Taiwan.

[4]Department of Physics, National Taiwan University, Taipei 10617, Taiwan.

*Correspondence and requests for materials should be addressed to H.H.L. (email: hhl@ym.edu.tw) or C.H.L. (email: clee@gate.sinica.edu.tw)

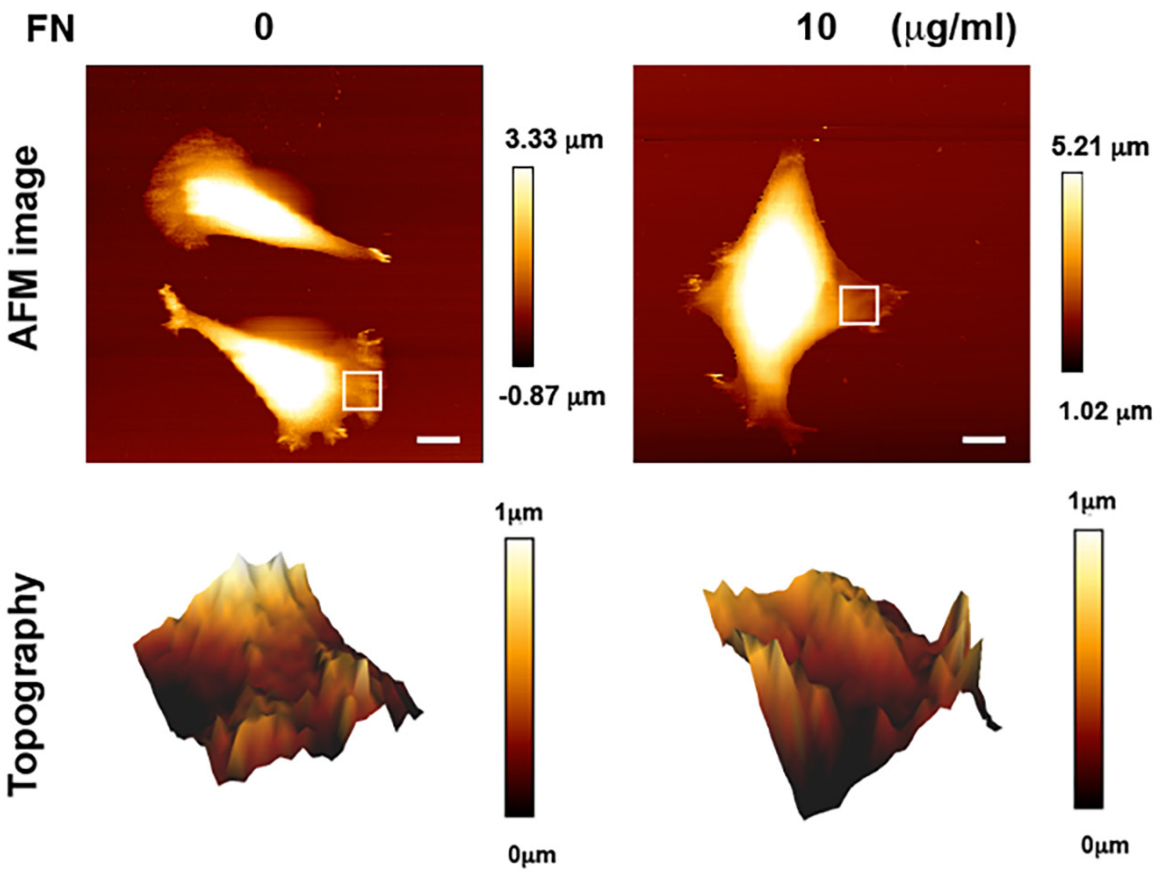

Supplementary Figure S1. Images of membrane topography determined by atomic force microscopy (AFM). MEFs were seeded on the polymer coverslip-bottom μ-dishes coated with 0 or 10 μg/ml FN for 6 hours for the measurement of membrane roughness by AFM. The regions marked by the white squares in the bright-field images are displayed in the membrane topography. Scale bar, 10 μm.

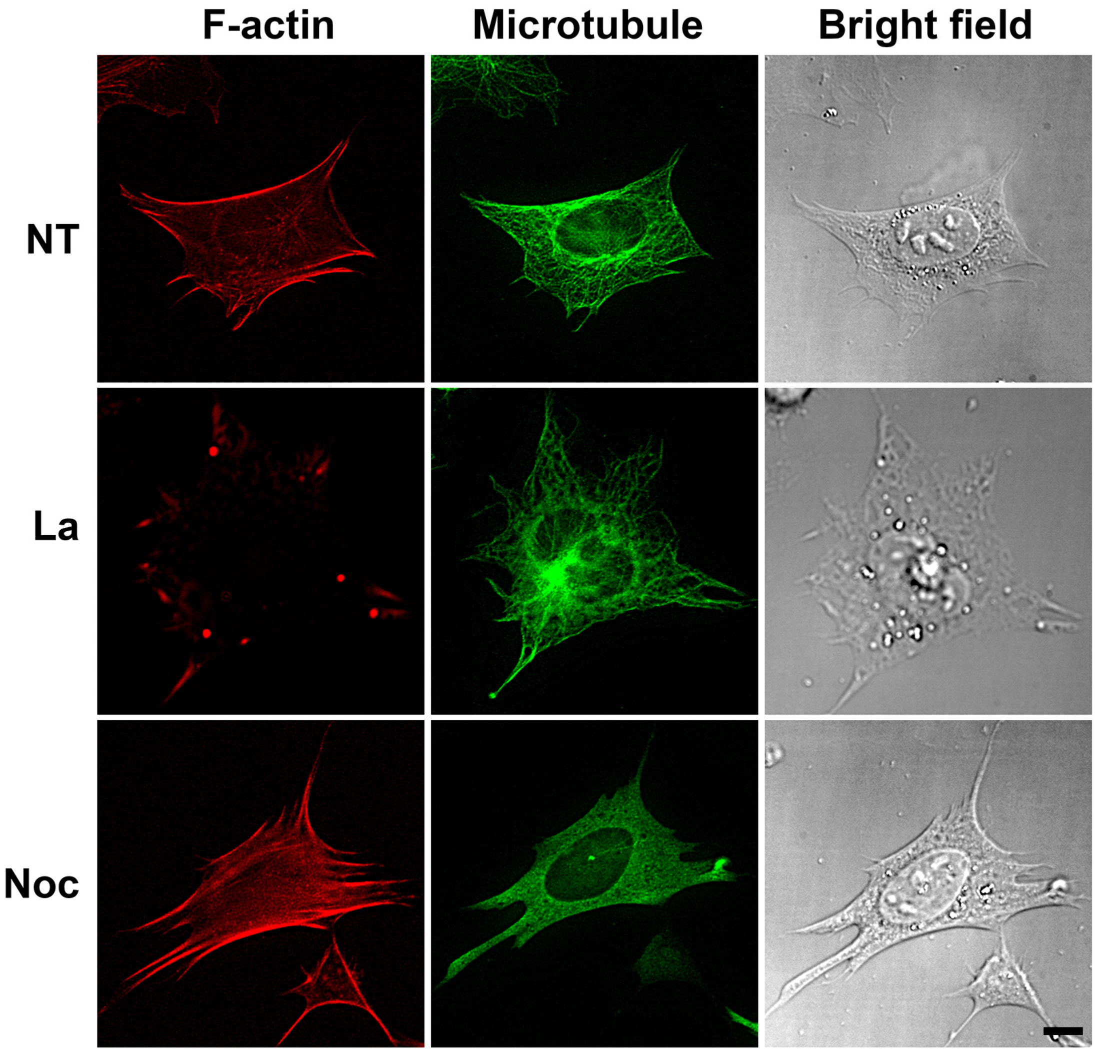

Supplementary Figure S2. Epi-fluorescence microscopy images of F-actin (red) and microtubules (green) in the MEFs with the treatment of 1.0 μM latrunculin (La) or 10 μM nocodazole (Noc) for 30 minutes. NT, no treatment. The fluorescence images confirm that La inhibited the polymerization of F-actin and Noc suppressed the formation of microtubules. The bright-field images show that the cell morphology was not varied significantly under the treatments. In order to improve the visibility of the cytoskeletons, we used Huygens Essential (Scientific Volume Imaging B.V., Hilversum, The Netherlands) to deconvolve the epi-fluorescence images. Scale bar, 10 μm.

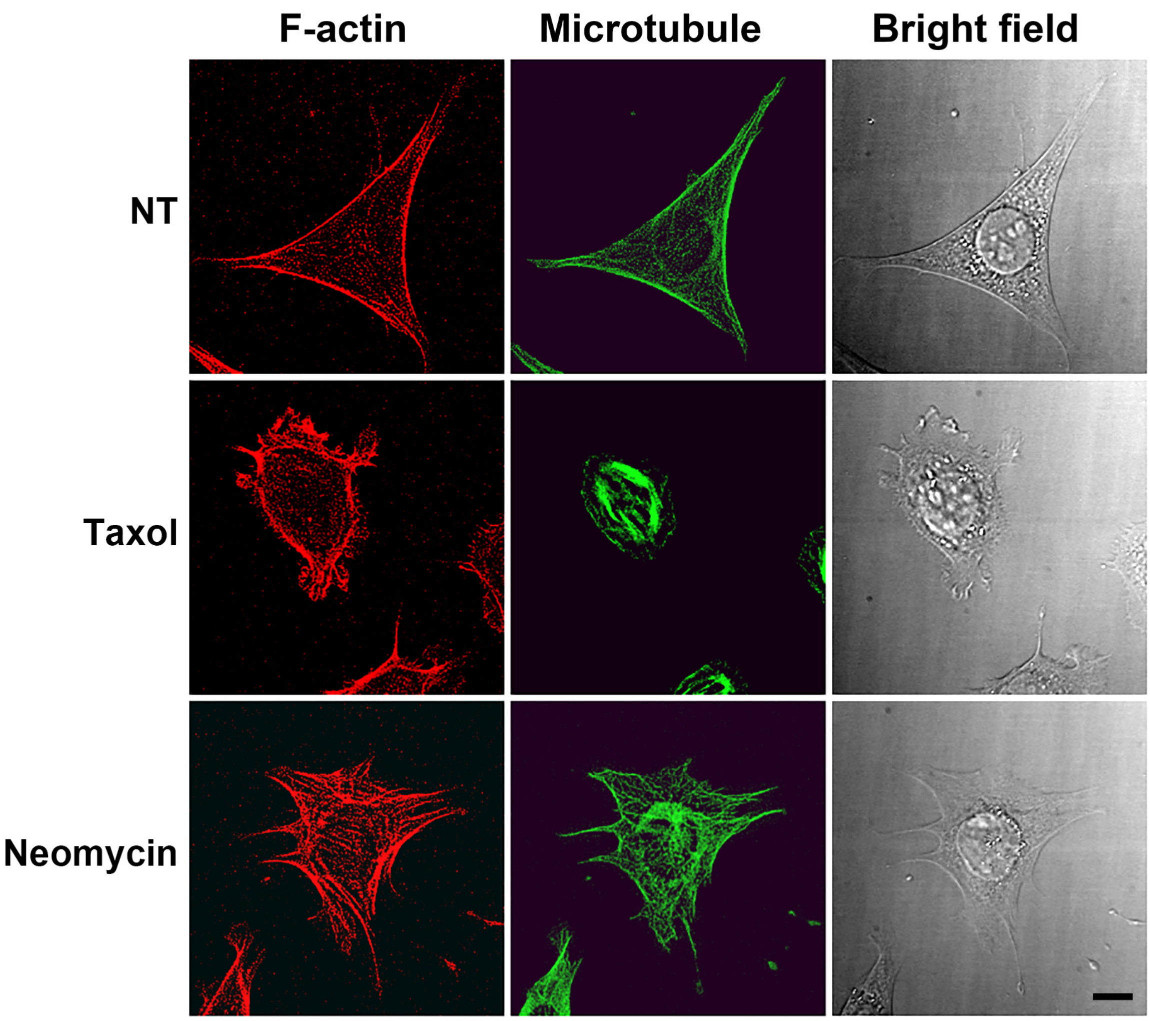

Supplementary Figure S3. Epi-fluorescence microscopy images of F-actin (red) and microtubules (green) in the MEFs with the treatment of 10 μM of Taxol for 4 hours or 3 mM of neomycin for 30 minutes. NT, no treatment. In order to improve the visibility of the cytoskeletons, we used Huygens Essential (Scientific Volume Imaging B.V., Hilversum, The Netherlands) to deconvolve the epi-fluorescence images. Scale bar, 10 μm.

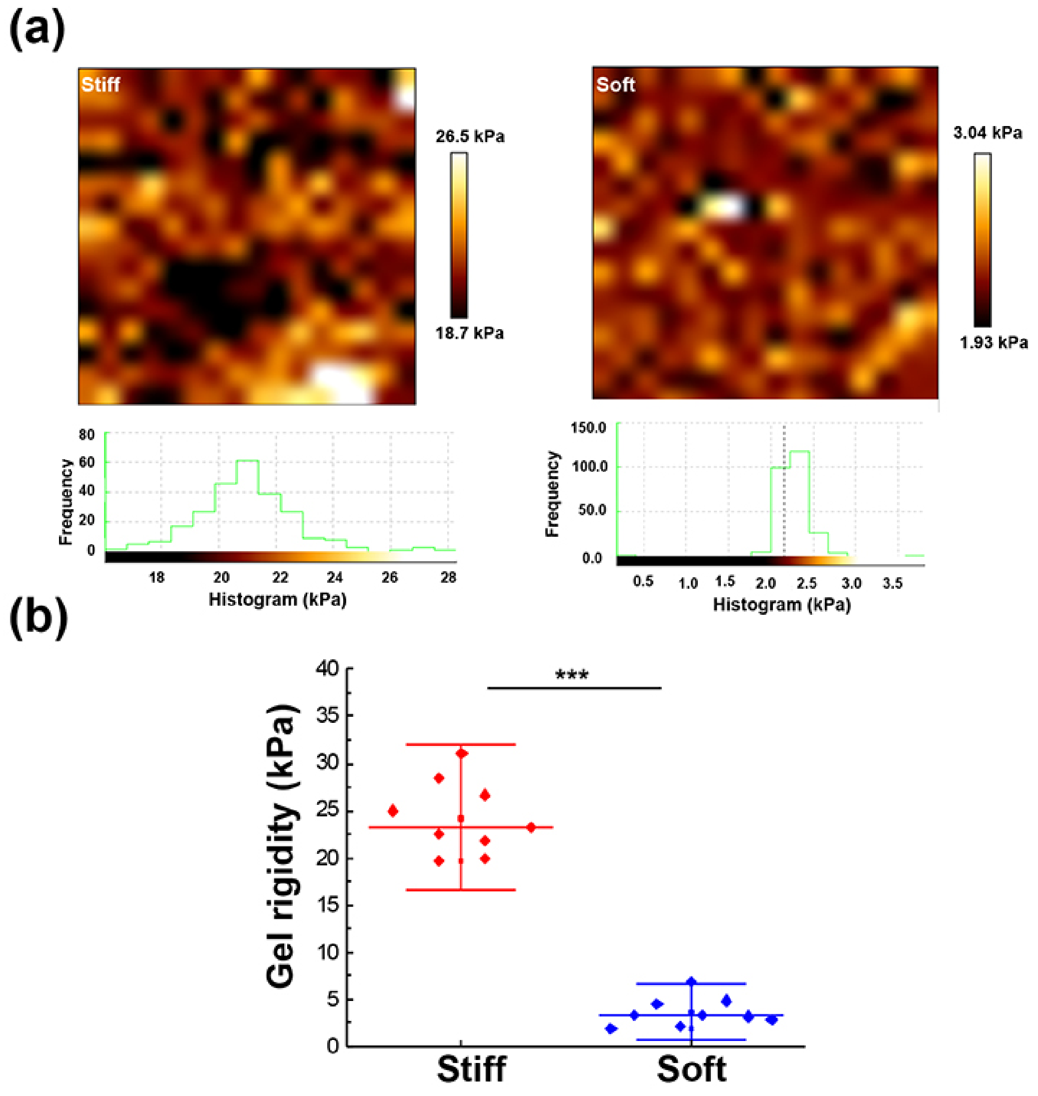

Supplementary Figure S4. Gel rigidity measured by AFM. (a) The rigidity maps and histograms of the stiff and soft gels. The side length of each map is 100 µm, and there are 16 × 16 measurement points in each map. (b) The rigidity distribution of 9 stiff and soft gel samples. On each sample we measured three arbitrarily selected 100 µm × 100 µm regions as shown in panel (a), and the average rigidity of the three selected regions is plotted as one point in this figure.

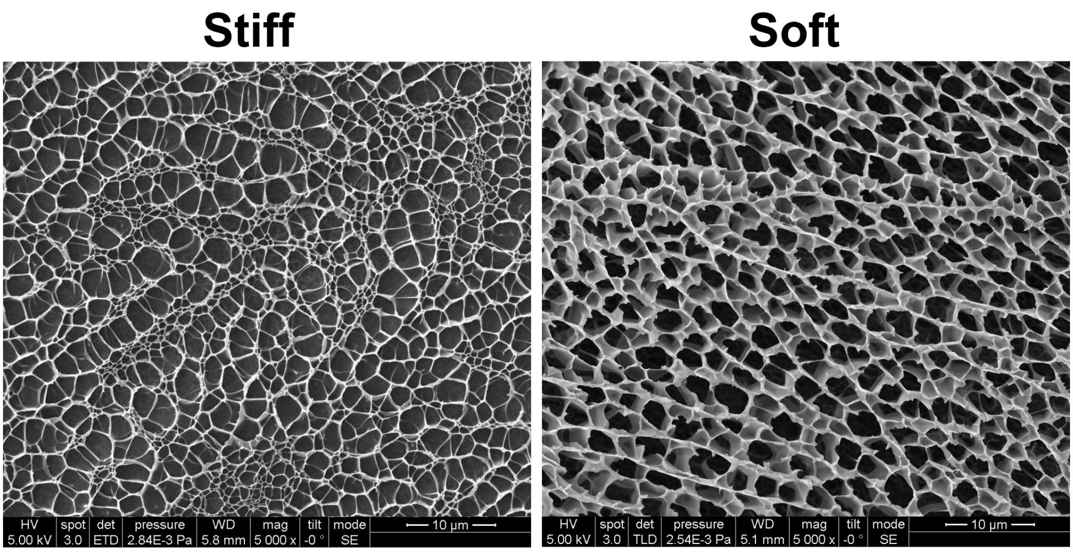

Supplementary Figure S5. Scanning electron microscopy (SEM) images of the PAA gel surface. The surface topography of stiff (24.2 kPa) and soft (3.7 kPa) PAA gels used in this study was determined by SEM. Briefly, hydrogel samples on 18 mm diameter coverslips were quench-frozen by plunging into melting propane cooled in liquid nitrogen. The surface was coated with 10 nm of gold and observed in a FEI Nova NanoSEM 200 at 5 kV. We quantified the distributions of pore areas in these two SEM images with ImageJ. On the stiff gel shown in this figure, the average pore area is 5.1 ± 19 μm^2. On the soft gel the average pore area is 3.3 ± 3.2 μm^2. Owing to the large variations, we could not claim that the pore sizes on the two gels were different.
